# Dysbiosis and genomic plasticity in the oily scalp microbiome: a multi-omics analysis of dandruff pathogenesis

**DOI:** 10.3389/fmicb.2025.1595030

**Published:** 2025-07-04

**Authors:** Han Yu, Jiayi Li, Yan Wang, Tongze Zhang, Tahir Mehmood, Olivier Habimana

**Affiliations:** ^1^Biotechnology and Food Engineering Program, Guangdong Technion-Israel Institute of Technology, Shantou, China; ^2^Faculty of Biotechnology and Food Engineering, Technion-Israel Institute of Technology, Haifa, Israel

**Keywords:** scalp microbiome, dysbiosis, dandruff, metagenomics, genomic plasticity, horizontal gene transfer, multi-omics

## Abstract

**Introduction:**

Dandruff, affecting ~50% of the global population, is a prevalent scalp condition linked to microbial dysbiosis and inflammation, significantly impacting quality of life.

**Methods:**

This study employed an integrative omics approach, utilizing 16S rRNA and ITS1 amplicon sequencing alongside shotgun metagenomics, to analyze the scalp microbiome of 65 individuals with varying scalp conditions (healthy oily, healthy non-oily, and dandruff oily).

**Results:**

Distinct microbial profiles were identified, with an increased abundance of pathogenic genera such as Staphylococcus in the dandruff oily (DO) group, contrasted with the presence of Cutibacterium in healthy cohorts.

**Discussion:**

Functional profiling revealed elevated DNA repair mechanisms in the DO group, indicative of stress stemming from pathogen overgrowth, while healthy non-oily samples demonstrated enhanced functions for scalp homeostasis. Notably, the increase in genomic plasticity in the DO group, characterized by antimicrobial resistance genes and mobile elements, underscores the complex interplay of microbial dynamics in dandruff pathology, advocating for microbiome-targeted therapies.

## 1 Introduction

Dandruff represents a persistent scalp concern that affects nearly half of the population at some stage in their lives (Borda and Wikramanayake, 2015). It significantly impacts individuals' quality of life, causing symptoms such as itching, flaking, and social embarrassment, particularly among young adults where incidence rates are notably high (Apetri and Gurgas, 2022; Seite et al., 2009; Zingkou et al., 2020). Although common, the exact reasons for dandruff are still not fully understood, which makes creating effective long-term treatments challenging (Limbu et al., 2021). Current therapies often rely on broad-spectrum antifungals and antibacterials, such as Ketoconazole, Selenium Sulfide, and Pyrithione Zinc, but typically provide only temporary relief, indicating persistent dysregulation within the scalp ecosystem (Vano-Galvan et al., 2024; Schwartz et al., 2011; Narshana and Ravikumar, 2018). The scalp hosts a diverse microbiome, including bacteria, fungi, and viruses, which are crucial for skin health (Mayser et al., 2024). This intricate ecosystem is influenced by environmental factors (such as air quality, temperature, humidity, and pH), host immune responses, and sebum production (Polak-Witka et al., 2020). Disruptions to this balance can cause dermatological conditions including dandruff, notably influenced by *Malassezia* species, especially *Malassezia restricta* (Meray et al., 2018; Saunders et al., 2012). However, its role is likely complex and intertwined with the overall composition and function of the microbial community (Mayser et al., 2024). While studies have consistently shown an overabundance of *Malassezia* in scalps affected by dandruff (Honnavar et al., 2024; Jourdain et al., 2023), viewing it as the sole causative agent oversimplifies the intricate interactions present within this ecological niche.

Improvements in sequencing technologies and bioinformatics are deepening our comprehension of the scalp microbiome. Nonetheless, many specific mechanisms leading to dandruff-related dysbiosis and its consequences for scalp health remain unclear. The etiology of dandruff is frequently associated with three genera, yet their interactions with other scalp microbes need further exploration. Genera including *Cutibacterium* and *Staphylococcus* have been recognized as the predominant bacterial species within the scalp microbiome (Watanabe et al., 2020). Notably, higher ratios of *Cutibacterium* are observed in healthy scalps, whereas elevated levels of *Staphylococcus* are found in dandruff-affected individuals (Tao et al., 2021). *In vitro* studies suggest that *Staphylococcus epidermidis* may inhibit other species, and evidence indicates potential beneficial interactions between *Cutibacterium acnes* and *Malassezia restricta* (Numata et al., 2024; Nakamura et al., 2020). The findings underscore a sophisticated network of microbial interactions, in which fluctuations in the abundance of one species can precipitate cascading effects throughout the entire system community.

Furthermore, previous research emphasizes the critical role of co-occurrence and co-exclusion networks between bacteria and fungi in maintaining scalp health (Wang et al., 2022). The study involving dandruff patients found that interactions among microbes and fungi on the scalp may significantly contribute to dandruff development. However, a comprehensive understanding of these complex relationships and the mechanisms regulating microbial community stability and resilience remains elusive.

This study adopts an integrative omics methodology to investigate the intricate interactions between the scalp microbiome and the pathogenesis of dandruff. We analyze the taxonomic and functional diversity of scalp microbiomes in healthy vs. dandruff-prone individuals through advanced sequencing and bioinformatics. By integrating data from various omics platforms, we aim to provide a thorough understanding of dandruff pathogenesis. This multifaceted approach, encompassing high-resolution taxonomic profiling, functional pathway analysis (utilizing KEGG, eggNOG, CAZy, and PHI), and assessing genomic plasticity, will yield valuable insights into the factors driving dandruff-associated dysbiosis. Ultimately, these findings will significantly contribute to developing targeted and effective therapeutic strategies that transcend current, often temporary solutions, paving the way for innovative treatment approaches aimed at restoring the ecological balance of the scalp microbiome and alleviating both the symptoms and long-term impacts of dandruff.

## 2 Materials and methods

### 2.1 Study population and sample collection

Sixty-five volunteers (33 females, 32 males) with diverse scalp conditions were recruited from Shantou and Putian, China. Participants filled out a questionnaire regarding hair washing frequency, scalp type, and self-assessed condition. Scalp samples were collected aseptically using sterile cotton swabs in conjunction with sterile combs. Each scalp area was thoroughly combed for 5 min, repeated three times, to maximize sample collection. Swabs were immediately immersed in 1 mL DNA/RNA Shield (Jianshibio, China) and stored at −20°C until further processing. The study protocol received ethical approval from Guangdong Technion-Israel Institute of Technology-E20231121001. All participants granted their informed consent before participation enrolment.

Participants were categorized into three groups based on self-assessment questionnaires and visual inspection by a trained dermatologist: (1) Dandruff Oily (DO): Individuals reporting visible dandruff flakes and exhibiting oily scalp conditions. (2) Healthy Oily (HO): Individuals reporting no visible dandruff flakes and exhibiting oily scalp conditions. (3) Healthy Non-Oily (HN): Individuals reporting no visible dandruff flakes and exhibiting non-oily scalp conditions. The exclusion parameters encompassed: any antecedent of cranial dermatoses (such as psoriasis or eczema), utilization of topical or systemic antifungal or antibiotic pharmacological agents within the preceding month, and any additional significant health afflictions that could influence the cutaneous microbiome. All participants supplied documented informed consent before registration. The visual inspection was conducted to objectively identify dandruff flakes.

### 2.2 DNA extraction and amplicon sequencing

Genomic DNA was extracted from samples using the ZymoBIOMICS DNA Microprep Kit (Zymo Research, USA) following the manufacturer's instructions, including a 10-min bead-beating step using an MX-C cell disruptor (Dlab, China) to ensure efficient cell lysis.

Amplicons for the bacterial 16S rRNA gene V4 region were generated using the primers GTGCCAGCMGCCGCGGTAA and GGACTACHVGGGTWTCTAAT. For the fungal ITS1 region, the primers used were CTTGGTCATTTAGAGGAAGTAA and GCTGCGTTCTTCATCGATGC. PCR reactions (15 μL final volume) employed Phusion^®^ High-Fidelity PCR Master Mix (New England Biolabs). PCR products were purified using magnetic beads (Agencourt AMPure, XP Beckman Coulter) and size-selected via gel electrophoresis. Illumina library preparation was performed according to the manufacturer's protocol, with quality assessment by AATI Fragment Analyzer and quantification using Qubit fluorometry and qPCR. Sequencing was performed on an Illumina NovaSeq 6000 platform using a paired-end 250 bp (PE250) protocol.

Raw reads were processed using FASTP (v0.23.1) for quality filtering and adapter trimming (Chen et al., 2018). Paired-end reads were merged using FLASH (v1.2.11) (Magoc and Salzberg, 2011). Amplicon sequence variants (ASVs) were identified using DADA2 within QIIME2 (v2022.2) (https://qiime2.org) (Callahan et al., 2016). The SILVA database was utilized for bacteria taxonomic assignment, while the UNITE database was used for fungi (Yilmaz et al., 2014; Abarenkov et al., 2024). Chimeric sequences were removed using QIIM2′s inbuilt chimera detection algorithm. In addition, to minimize host DNA contamination of the samples, all the metagenomic raw reads were screened and the reads matched to human genome in NCBI database were discarded and removed.

### 2.3 Shotgun metagenomic sequencing

Seven samples per group (HN, HO, DO) were selected from the full cohort (n = 65) for shotgun metagenomic sequencing, matched by age (± 3 years), sex, geographic origin, and scalp condition. DNA from these samples was pooled per group. These seven samples per group were selected to balance statistical power with sequencing costs, as preliminary power analyses suggested this would capture major trends. DNA was fragmented to ~350 bp using a Covaris ultrasonic disruptor. Illumina library preparation and sequencing were performed using a PE150 protocol on the NovaSeq 6000 platform. Raw reads were processed using FASTP (v0.23.1) for quality filtering and adapter trimming. High-quality reads were assembled using MEGAHIT (Li et al., 2015).

### 2.4 Bioinformatic analysis

#### 2.4.1 Amplicon sequencing data analysis

Beta diversity group differences were tested using Kruskal–Wallis on per-sample centroid distances (mean distance to group centroid), a non-parametric alternative to PERMANOVA for small sample sizes. PCoA and ANOSIM were used to visualize and confirm group separation. Furthermore, data visualization included a genus-level correlation network performed in R (https://www.R-project.org/). Functional predictions for bacterial communities were made using PICRUSt (v1.1.4) (Langille et al., 2013). For co-occurrence network analysis, we employed the SparCC algorithm implemented in the R package “SpiecEasi” (Kurtz et al., 2015). This algorithm is designed to infer sparse correlations in compositional data. Using Cytoscape (version 3.9.1), we generated interactive network visualizations that enable exploration and manipulation of network structures rooted in node and edge properties.

#### 2.4.2 Shotgun metagenomic data analysis

Open reading frames (ORFs) were predicted from scaffolds longer than 500 bp using MetaGeneMark (Zhu et al., 2010). Taxonomic annotation of unigene sequences was conducted by aligning them with the Micro_NR database using DIAMOND software (Buchfink et al., 2015). The abundance tables at each taxonomic level were visualized using Krona analysis (Ondov et al., 2011). For functional annotation, predicted proteins were aligned against several databases, including KEGG, eggNOG, CAZy, VFDB, and PHI, utilizing DIAMOND (Kanehisa et al., 2006; Huerta-Cepas et al., 2016; Cantarel et al., 2009; Chen et al., 2005; Urban et al., 2022; Buchfink et al., 2015). Resistance genes were identified using the RGI tool and compared with the CARD database (McArthur et al., 2013; Alcock et al., 2020). To investigate mobile genetic elements (MGEs), unigene sequences were compared with insertion sequences (ISs) via the ISfinder database (Siguier et al., 2006), integrons via the Integrall database (Moura et al., 2009), and plasmids via the PLSDB database (Galata et al., 2019). Data visualization included abundance histograms and heatmaps generated using R, utilizing Bray-Curtis distance clustering for both functional pathways and taxa through the “hclust” function with the Ward D2 linkage method. Heatmap values were represented as *Z-*scores, calculated as the difference between a sample's relative abundance and the category average, divided by the standard deviation.

## 3 Results

### 3.1 Microbial community composition of healthy and dandruff scalp

High-throughput sequencing of the 16S rRNA gene V4 region yielded an average of 118,498 raw reads per sample (*n* = 65), while ITS1 region sequencing generated 54,612–154,544 reads per sample (mean = 107,198 reads). The top ten bacterial phyla and thirty most abundant genera are detailed in Supplementary Table S1. Analysis showed *Actinobacteriota* as the main bacterial phylum in all samples, followed by *Firmicutes*. Three dominant genera were *Cutibacterium, Staphylococcus*, and others *Lawsonella*. Fungal communities were predominantly *Basidiomycota* (98% average), with *Malassezia* and *Malasseziales_gen_Incertae_sedis* as the most prevalent genera.

**Figure 1:**
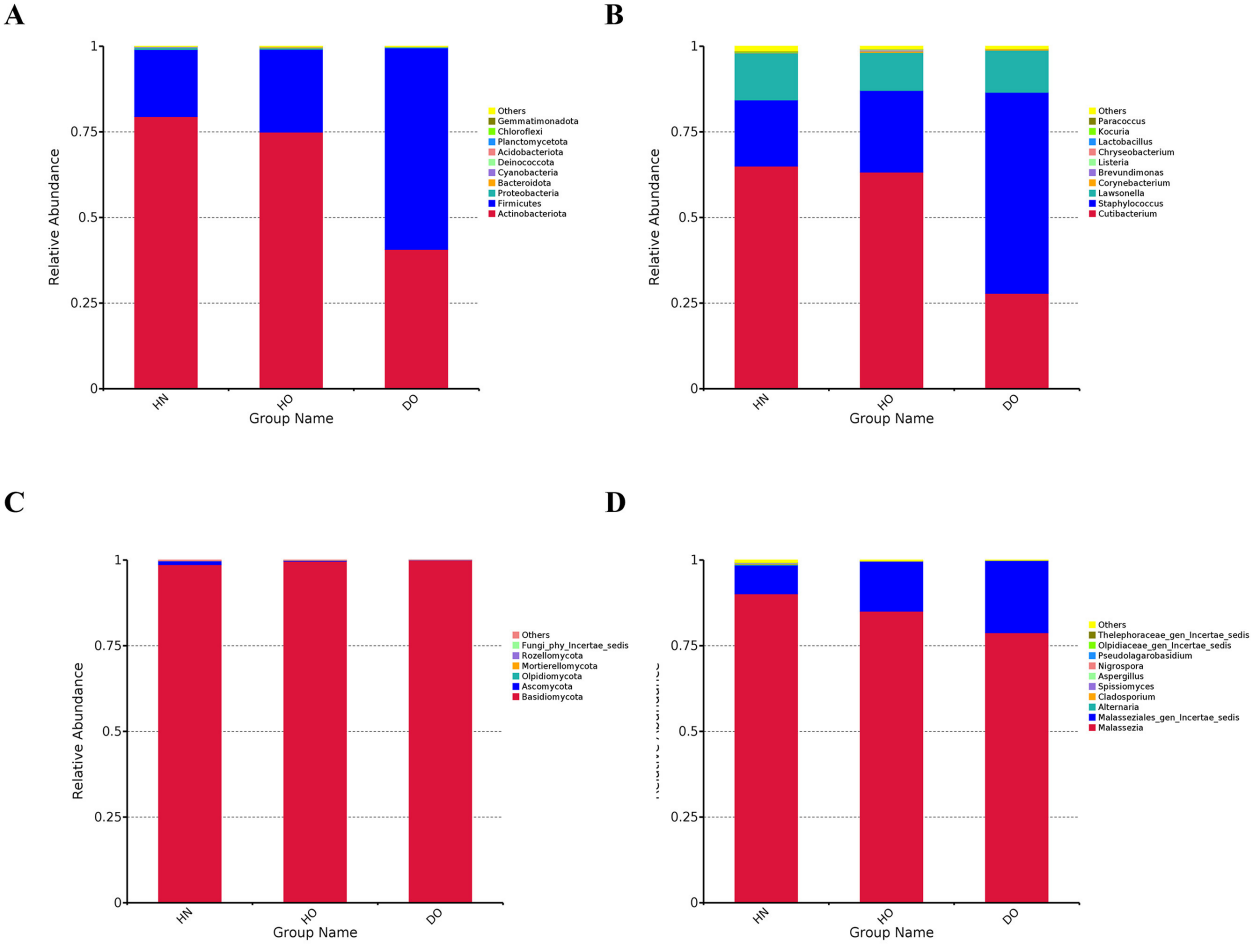
Taxonomic annotations at the phylum and genus levels for bacterial and fungal communities. Histograms for bacterial phylum **(A)** and genus-level annotations for bacteria **(B)**, as well as histograms for fungal phylum **(C)** and genus-level annotations for fungi **(D)**.

To investigate microbiome differences across scalp conditions, samples were categorized into three groups: healthy non-oily (HN), healthy oily (HO), and dandruff oily (DO), each comprised of seven samples. *Actinobacteriota* was more abundant in HN (79.45%) and HO (74.94%) than DO (40.65%, *p* < 0.0001). Conversely, *Firmicutes* were more abundant in DO (58.85%) than HN (19.63%) and HO (24.22%, *p* < 0.0001) (Figure 1A). Other phyla exhibited low relative abundance across all groups. This significant shift in the relative abundance of dominant bacterial phyla suggests a substantial alteration in the microbial ecosystem between healthy and dandruff-affected scalps.

Genus-level analysis (Figure 1B) revealed further distinctions. *Staphylococcus* was more abundant in the DO group (58.70%) than in HN (19.35%) and HO (23.85%, *p* < 0.0001). In contrast, *Cutibacterium* was significantly more abundant in HN (64.98%) and HO (63.24%) groups compared to DO (27.87%, *p* < 0.0001). *Corynebacterium* was more abundant in HO (0.43%) and DO (0.41%) than in HN (0.19%). These genus-level variations suggest a potential role for these bacterial taxa in dandruff development or progression.

Fungal analysis (Figure 1C) showed *Basidiomycota* as the dominant phylum (>98%) in all groups. *Ascomycota* was more prevalent in HN (1.14%) than in HO (0.18%) and DO (0.08%). *Rozellomycota, Mortierellomycota*, and *Olpidiomycota* were present at low abundances across all groups. While significant differences were observed at the fungal genus level (Figure 1D), these variations were less pronounced compared to the observed bacterial shifts. *Malassezia* represented the majority of fungal communities across all groups (90.14%, 85.05%, and 78.80% in HN, HO, and DO, respectively). The DO group exhibited a higher relative abundance of *Malasseziales_gen_Incertae_sedis* (21.10%) compared to HN (8.36%) and HO (14.62%).

The composition of the overall taxonomy and abundance patterns resulting from the 16S rRNA and ITS1 amplicon sequencing (Figure 1) largely agreed with findings from shotgun metagenomic sequencing (Supplementary Figure S1). Both methodologies indicated a substantial increase in *Staphylococcus* and a decrease in *Cutibacterium* abundance within the DO group compared to the healthy cohort.

### 3.2 Diversity indices and community comparisons of scalp microbiota

**Figure 2:**
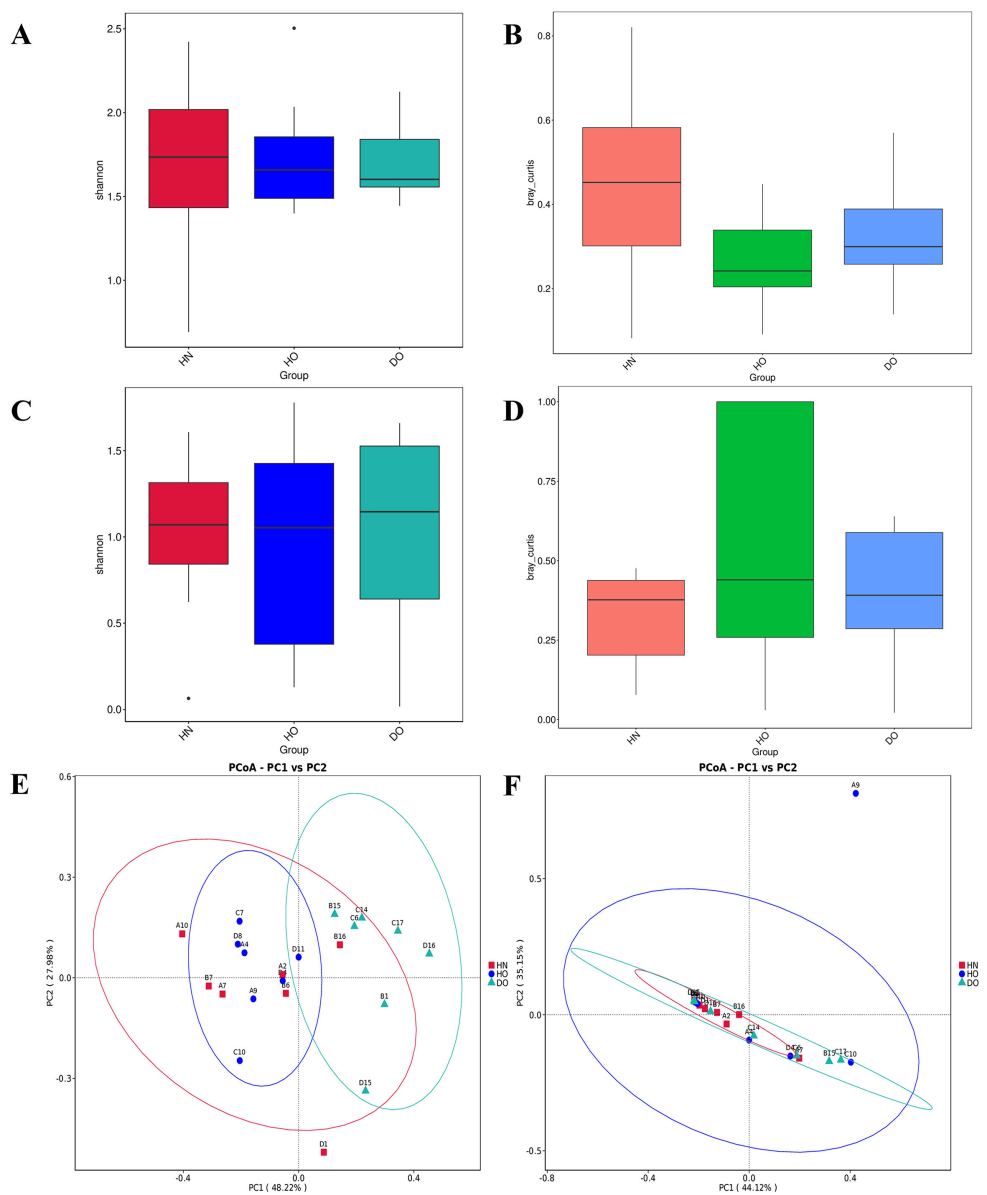
**(A)** Bacterial Shannon alpha diversity box plots with pairwise Wilcoxon rank-sum tests. **(B)** Box plots of inter-group median Bray-Curtis dissimilarities (computed as the average distance of each sample to all samples in the opposing group). Statistical significance was evaluated utilizing Kruskal–Wallis tests (3-group comparison) succeeded by pairwise Wilcoxon tests (FDR-adjusted). **(C)** Fungal Shannon alpha diversity box plots with pairwise Wilcoxon rank-sum tests. **(D)** Box plots of between-group median unweighted UniFrac distances, analyzed as in **(B)**. **(E, F)** PCoA plots of Bray-Curtis dissimilarities for bacterial **(E)** and fungal **(F)** communities (Qiime2-emperor), with axes showing variance explained (%). Confidence ellipses denote groups with >3 samples. Beta diversity box plots (*B*/*D*) reflect group-wise dissimilarity medians (see Methods). While PERMANOVA is standard for distance matrices, Kruskal-Wallis was used here as a non-parametric alternative for median distance comparisons.

Alpha diversity analysis assessed within-group diversity using Chao1, observed operational taxonomic units (OTUs), dominance, Good's coverage, Pielou's evenness, Simpson's, and Shannon diversity indices (Supplementary Table S1). No significant differences in alpha diversity existed among the three groups (HN, HO, DO), indicating similar species richness and evenness for bacterial and fungal communities (Figures 2A, C).

Beta diversity analysis using Bray-Curtis dissimilarity compared microbial community structures across groups. Bacterial community structure differences were observed between groups (*p* < 0.05, Figure 2B). This contrasted with the fungal communities, where no statistically significant differences were found across groups (Figure 2D).

Principal coordinate analysis (PCoA) of Bray-Curtis dissimilarities from 16S rRNA data (Figure 2E) showed a clear separation between healthy groups (HN, HO) and the dandruff oily (DO) group. Analysis of similarities (ANOSIM) confirmed significant differences in bacterial community structure between DO and both HN (*p* = 0.015) and HO (*p* = 0.005). This contrasted with the fungal communities, where no statistically significant differences were found across groups (Figure 2D).

**Figure 3:**
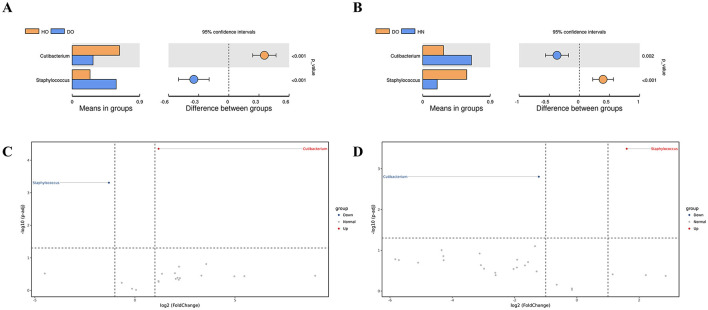
Group differences identified via *t-*test. **(A)** Comparison between HO and DO, **(B)** comparison between HN and DO, **(C)** volcano plot for HO vs. DO, and **(D)** volcano plot for HN vs. DO, with red (upregulated) and blue (downregulated) dots indicating differential genus abundance.

Pairwise comparisons of bacterial genera was conducted through genus-level *t-*tests (Figures 3A, B). The results were further illustrated using volcano plots (Figures 3C, D), which combined *p*-values with fold changes in abundance. A noteworthy difference in the average abundance of *Cutibacterium* and *Staphylococcus* was found between the HO and DO groups (*p* < 0.001 for both genera). *Cutibacterium* was significantly more abundant in the HO group, while *Staphylococcus* predominated in the DO group. The volcano plot (Figure 3C) confirmed this, showing a substantial positive fold change [–log10(*p*-adj) > 4] for *Cutibacterium* in HO relative to DO, indicating strong upregulation in healthy oily scalps. Conversely, *Staphylococcus* showed a significant negative fold change in HO compared to DO. Comparative analyses of the DO and HN groups (Figure 3B) indicated a notably higher prevalence of *Staphylococcus* in the DO group (Figure 3D). While *Cutibacterium* showed numerically lower abundance in DO compared to HN, the difference was not statistically significant.

### 3.3 PICRUSt analysis of microbial functions and network dynamics in microbial communities

**Figure 4:**
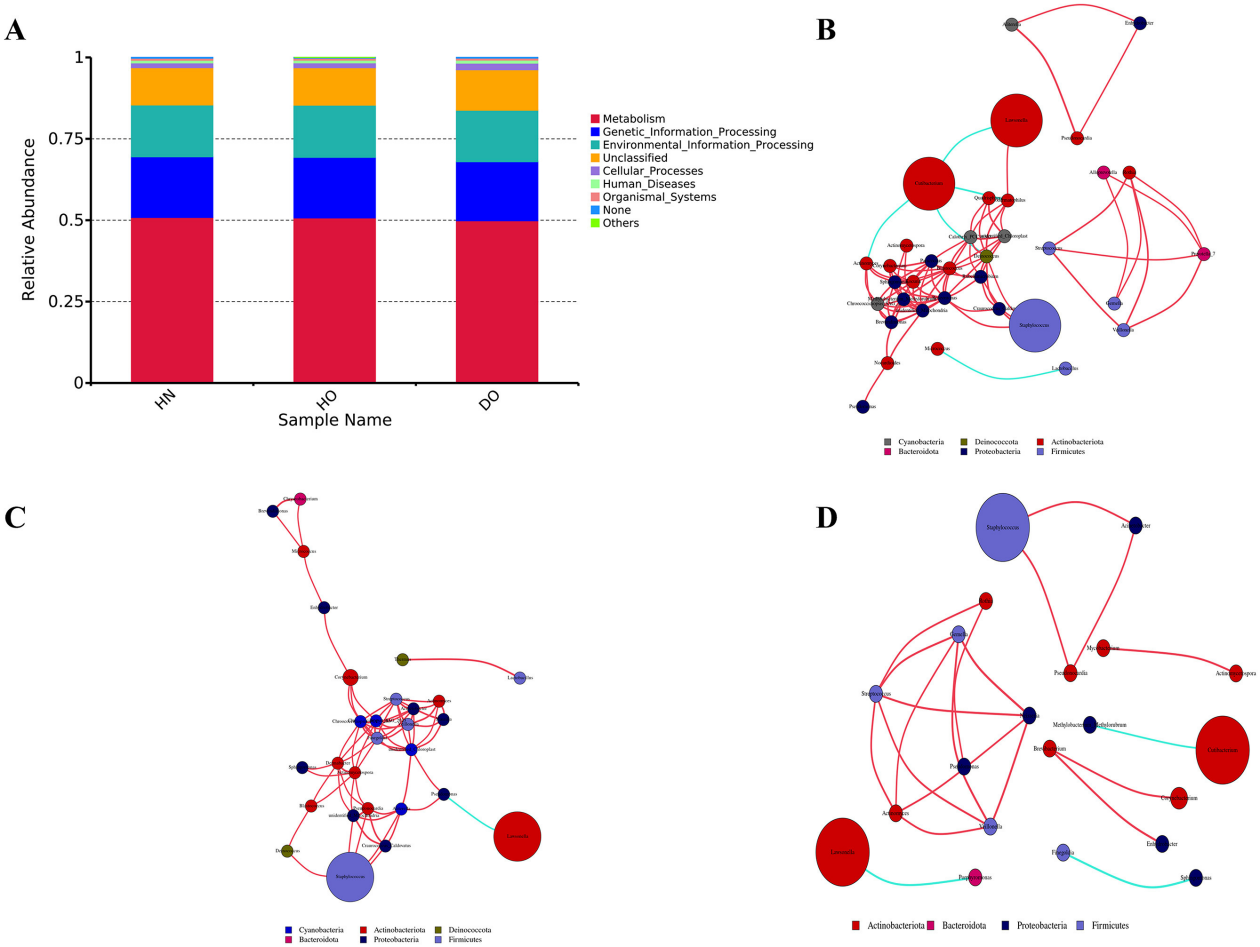
PICRUSt functional predictions and genus-level network analysis. **(A)** Functional prediction histogram. Network analysis for HN **(B)**, HO **(C)**, and DO **(D)** groups, with node size representing relative abundance and color of line indicating positive (red) or negative (blue) correlations.

Functional profiles were predicted using PICRUSt2, which inferred pathways from 16S rRNA gene sequencing and the KEGG database. Metabolic pathways made up about 50% of the predicted functions: 50.87% (HN), 50.72% (HO), and 49.84% (DO). Genetic Information Processing constituted the second most prevalent category, comprising 18.65%, 18.63%, and 18.15% for the HN, HO, and DO groups, respectively. This was succeeded by Environmental Information Processing, which accounted for 15.91%, 16.01%, and 15.81% for HN, HO, and DO, correspondingly. These three functional categories represent the primary areas of predicted functional enrichment in the microbial communities across the three scalp groups (Figure 4A). In our analysis, the functional predictions from DIAMOND alignment (Supplementary Table S1) and PICRUSt2 were broadly consistent and both methods showed similar trends.

To examine inter-genus relationships, co-occurrence networks at the genus level were established for each respective genus group. Distinct interaction patterns emerged across groups. Within the HN group, the predominant genera, namely *Cutibacterium, Staphylococcus*, and *Lawsonella*, demonstrated intricate interrelationships with various other genera, as depicted in Figure 4B. A negative correlation between *Cutibacterium* and *Lawsonella* suggests potential competitive or inhibitory interactions. The HO group displayed similar interaction patterns but also included *Pseudonocardia* within its core network, and a negative correlation was observed between *Lawsonella* and *Pseudomonas*, a notable difference from the HN group (Figure 4C). In the DO group, both *Cutibacterium* and *Lawsonella* showed individual negative correlations with other genera (*Methylobacterium-Methylorubrum* and *Porphyromonas*, respectively). *Pseudomonas* exhibited extensive connections with various genera, including *Streptococcus*, highlighting its significant ecological role. Positive correlations were observed between *Staphylococcus* and *Pseudonocardia* (*p* = 0.005), as well as *Acinetobacter* (*p* = 0.029). The absence of correlations between *Staphylococcus* and *Cutibacterium* or *Lawsonella* in the DO group suggests niche differentiation or specialization (Figure 4D).

### 3.4 Taxonomic differences at the species level

**Figure 5:**
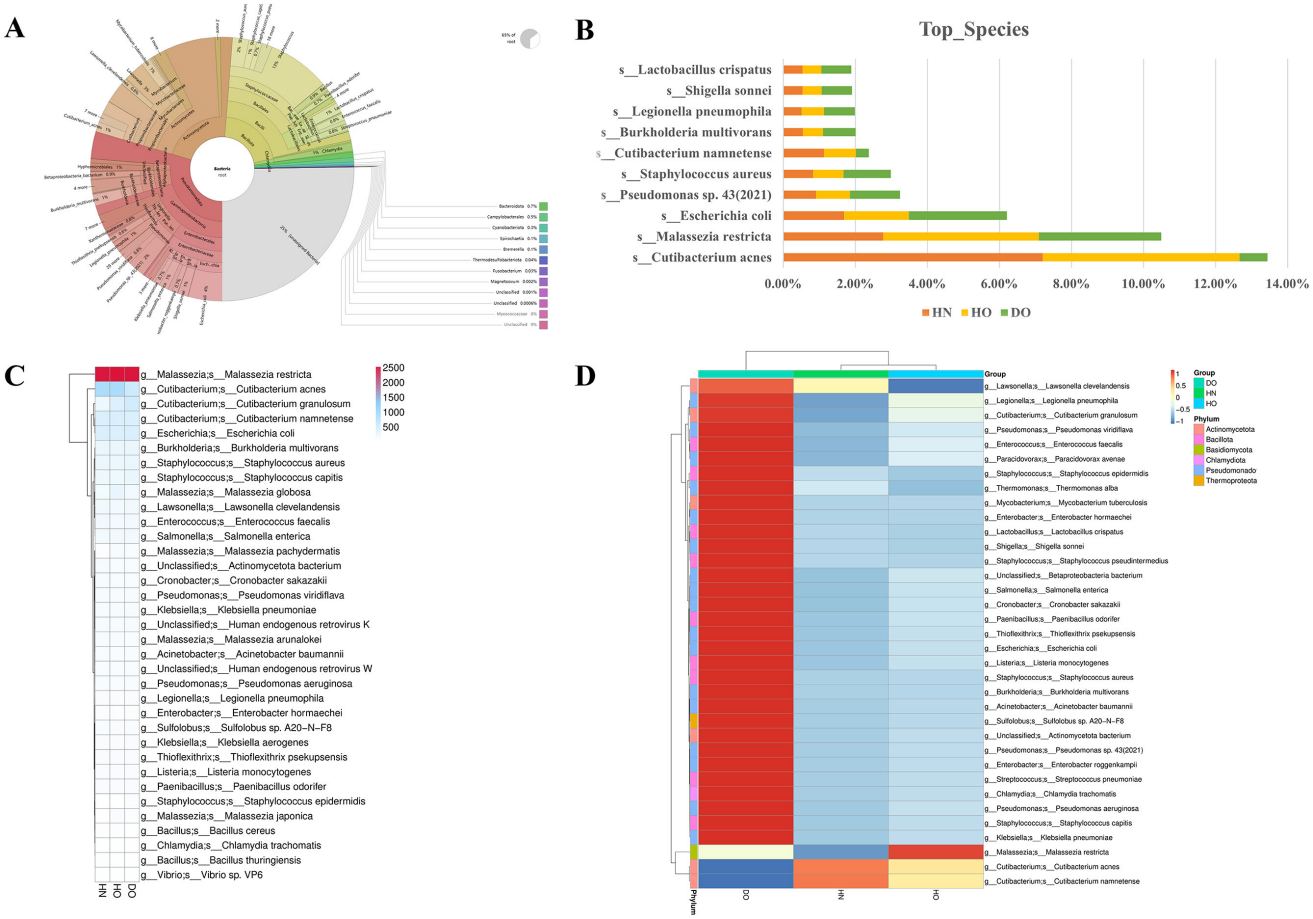
Species diversity between dandruff and healthy samples. Krona visualization for DO samples **(A)**; bar chart showing species abundance across HN, HO, and DO groups **(B)**. Heatmap of absolute unigene sequence counts (range 500–2,000) and abundance clustering for HN, HO, and DO **(C)**; genus-species level differential expression via clustering heatmap **(D)**, with color intensity reflecting *Z-*scores.

Shotgun metagenomic sequencing revealed notable differences in the scalp microbiome composition between healthy individuals and those affected by dandruff individuals. Taxonomic annotation, visualized using Krona (Supplementary Figure S1), provided a high-level overview of taxonomic distribution at the phylum and class levels. The dandruff oily cohort showed a bacterial composition comprising 65% of the microbiome, with increased *Staphylococcus* and decreased *Cutibacterium* abundance (Figure 5A).

Species-level analysis (Figure 5B) highlighted specific taxa differentiating the groups. *Cutibacterium acnes* showed a markedly higher prevalence in the HN (7.21%) and HO (5.45%) groups compared to the DO group (0.78%). Similarly, *Cutibacterium namnetense* showed higher relative abundance in HN (1.13%) and HO (0.89%) compared to DO (0.36%). In contrast, the DO group showed a higher prevalence of *Staphylococcus aureus* at 1.31%, compared to HN and HO. *Malassezia restricta*, frequently associated with dandruff, exhibited higher abundance in the HO (4.32%) and DO (3.39%) groups relative to HN (2.78%). *Lactobacillus crispatus*, recognized for its probiotic properties, was more prevalent in the DO group (0.83%) compared to HN and HO. These variations in species abundance may significantly influence alterations in skin barrier function observed in individuals with dandruff.

Further analysis involved a statistical heatmap of unigene annotation numbers and a species abundance clustering heatmap (Figures 5C, D) to visualize differences in microbial composition across the samples. *Malassezia restricta* exhibited consistently high unigene abundance across all groups (2,497, 2,526, and 2,510 in HN, HO, and DO, respectively). Conversely, *Cutibacterium acnes* abundance was significantly higher in HN (887) and HO (893) groups compared to DO (565). The species abundance heatmap (Figure 5D) indicated that the DO group exhibited greater abundances of *Staphylococcus, Pseudomonas*, and *Cutibacterium granulosum*. In contrast, *Cutibacterium acnes* and *Cutibacterium namnetense* were more prevalent in the HN group compared to the HO and DO groups. *Malassezia restricta* also showed greater relative abundance in HO compared to HN and DO.

### 3.5 Functional profiling of the scalp microbiome via KEGG pathway analysis

**Figure 6:**
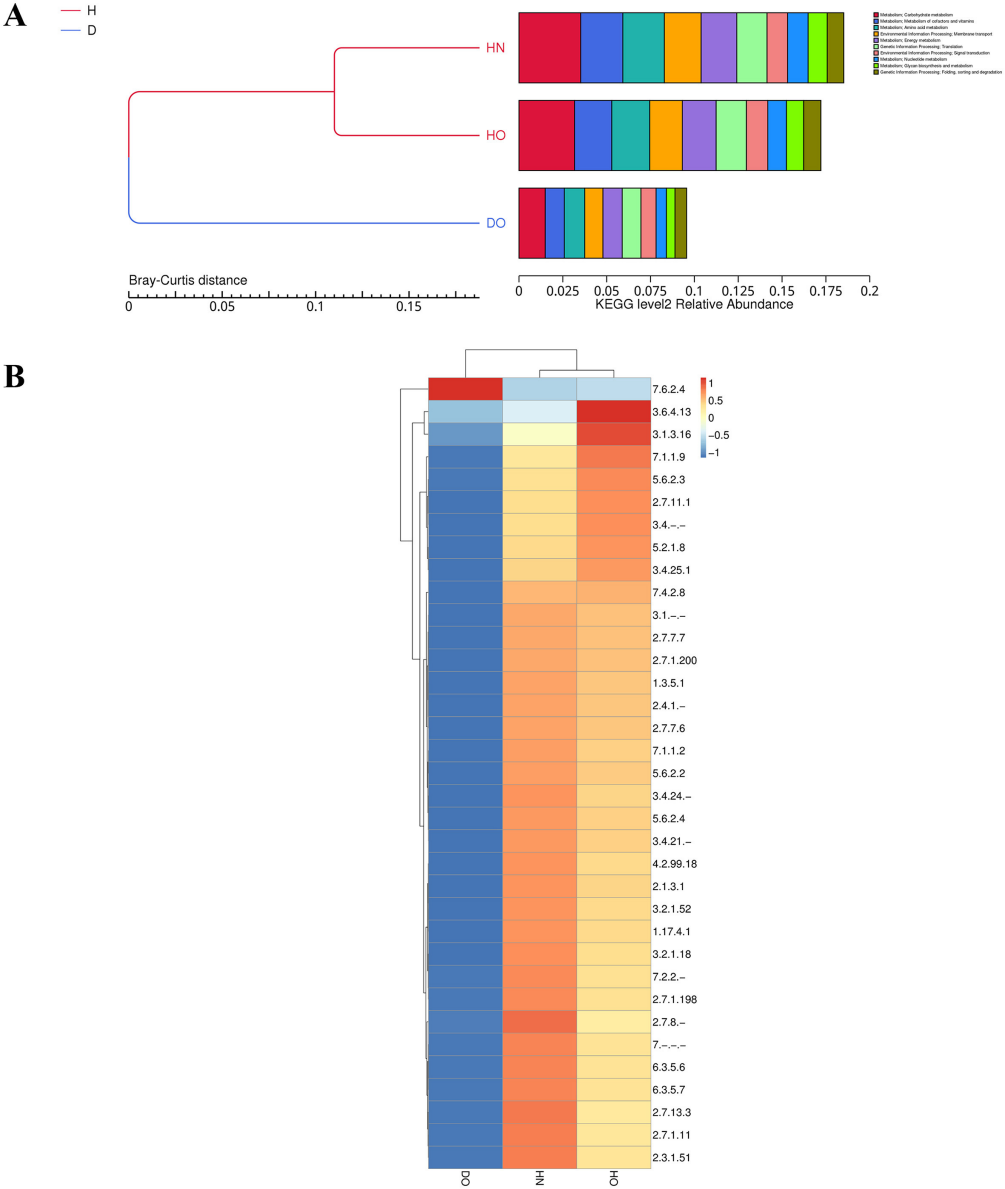
Bray-Curtis clustering and KEGG functional abundance distribution. Bray-Curtis distance clustering and functional relative abundance for level 2 KEGG categories **(A)**. Hierarchical heatmap of EC categories among healthy and dandruff groups **(B)**, with color intensity based on *Z-*scores.

Shotgun metagenomic sequencing data allowed for the investigation of the functional potential of the scalp microbiome across the three groups (HN, HO, DO). Level 1 KEGG pathway analysis, consistent with previous PICRUSt2 predictions (Supplementary Figure S2), revealed distinct functional profiles. Bray-Curtis hierarchical clustering (Figure 6A) indicated that HN and HO samples were functionally similar. Compared to DO, HN, and HO groups showed higher relative abundance of carbohydrate/amino acid metabolism, nucleotide/energy metabolism, and membrane transport pathways (though statistical testing was precluded by sample pooling). Furthermore, HN and HO groups showed greater representation of nucleotide and energy metabolism pathways compared to the DO group. The “Environmental Information Processing: Membrane Transport” KEGG category was significantly enriched in both HN and HO groups, suggesting differences in nutrient acquisition and environmental responsiveness between these groups and the DO group. Similarly, the “Genetic Information Processing: Translation” pathway was significantly enriched in HN and HO groups.

To further refine the functional analysis, Enzyme Commission (EC) numbers were annotated to identify specific enzymes. Hierarchical clustering of EC numbers (Figure 6B) showed that EC number 7.6.2.4, linked to ABC-type fatty-acyl-CoA transporters, was mainly found in the DO group, indicating a possible role in fatty acid transport. In contrast, EC numbers 3.6.4.13 (an ATPase linked to energy metabolism and transport) and 3.1.3.16 (a phospholipase connected to lipid metabolism and membrane dynamics) were significantly more common in the HO group.

### 3.6 Functional profiling of the scalp microbiome via eggNOG analysis

**Figure 7:**
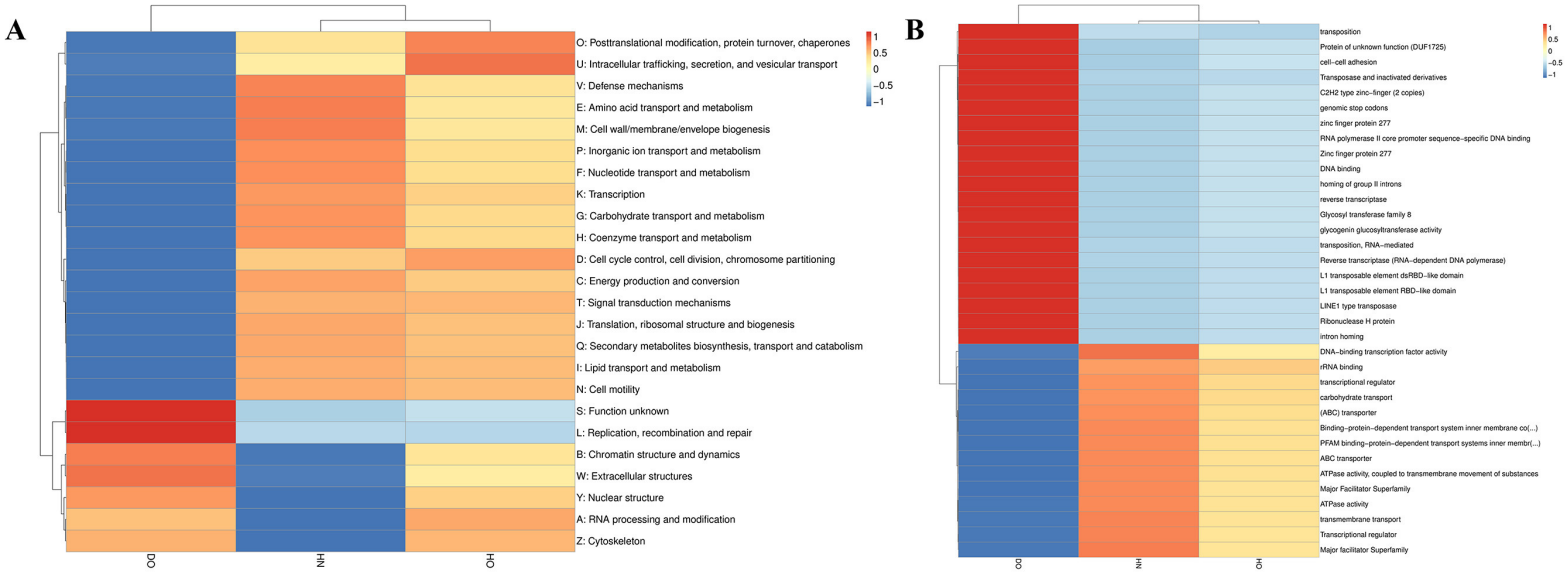
Functional profiling via eggNOG analysis. Functional categories at level 1 **(A)** and level 2 **(B)** visualized by hierarchical clustering heatmaps, with color intensity based on *Z-*scores.

Orthologous gene clusters were analyzed across scalp groups using the EggNOG database to assess functional differences. Hierarchical clustering (Figure 7A) showed that the DO group displayed increased activity in functions associated with replication, recombination, and DNA repair, indicating improved genomic maintenance and stability. In contrast, the HN group showed greater activity in transport and metabolism pathways, particularly membrane biogenesis, reflecting adaptations for nutrient uptake and cellular integrity. The HO group showed notable activity in post-translational modification, protein turnover, chaperone functions, secretion, vesicular transport, and cell cycle regulation. These variations highlight the distinct ecological roles and adaptive strategies of microbial communities within each scalp group.

Further analysis using level 2 hierarchical clustering (Figure 7B) revealed more specific functional enrichments. The DO group showed significant enrichment in transposition-related functions, with abundant transposases and transposable element-associated domains (L1, LINE1), indicating a high degree of genomic plasticity. The HN group displayed significant enrichment in carbohydrate transport functions, primarily via ABC transporters and the major facilitator superfamily, suggesting efficient carbohydrate metabolism and utilization. The HO group exhibited a more balanced functional profile, with moderate representation of both transposition-related and carbohydrate transport functions, along with rRNA binding and transcriptional regulation, consistent with a more generalist metabolic strategy compared to the HN and DO groups.

### 3.7 Function and interaction profilings referencing CAZy, VFDB, and PHI databases

**Figure 8:**
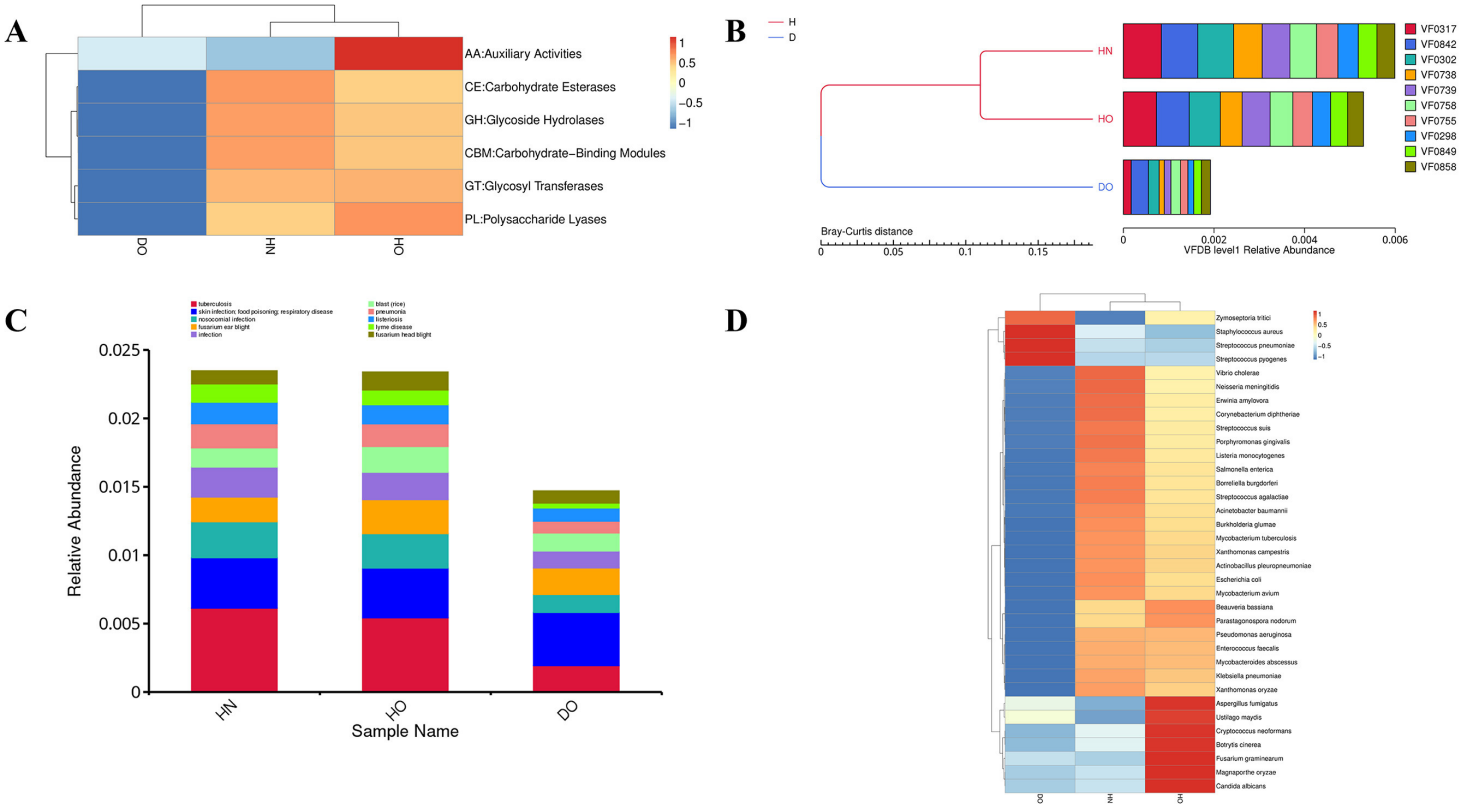
Functional profiling of CAZy, VFDB, and PHI categories. Hierarchical clustering heatmap of CAZy family abundances **(A)**; virulence factor clustering based on Bray-Curtis dissimilarity **(B)**; PHI level 1 abundance histogram **(C)** and level 2 hierarchical clustering heatmap **(D)**. Heatmap intensity represents *Z-*scores.

Hierarchical clustering of CAZyme family abundances (Figure 8A) revealed distinct functional adaptations across the three groups, potentially correlating with sebum composition and dandruff pathogenesis. The DO group showed a relatively lower abundance of polysaccharide lyases (PLs), suggesting reduced capacity for complex polysaccharide degradation. The HN group showed more glycosyl hydrolases (GHs), especially for degrading simple sugars from host-derived (skin cells) and environmental sources (e.g., skincare products). Increased levels of carbohydrate esterases (CEs) in the HN group, and comparable levels of glycosyl transferases (GTs) in both HN and HO groups, suggest enhanced capabilities for utilizing lipid-associated carbohydrates and modifying host-produced lipids. The HO group showed increased relative abundance of auxiliary activities (AAs) and PLs, indicating a greater capacity for complex polysaccharide degradation, which may contribute to the breakdown of stratum corneum components, potentially mitigating barrier disruption and inflammation associated with dandruff.

Hierarchical clustering utilizing Bray-Curtis dissimilarity (Figure 8B) showed that the healthy groups (HN and HO) exhibited higher community cohesion and resilience compared to the DO group. Although essential virulence factors (VF0317, VF0842, and VF0302) existed in every group, they were found to be more plentiful in the healthy groups. The higher abundance of VF0842 in the DO group suggests a specific role in dandruff pathogenesis. Other virulence factors (VF0738, VF0739, VF0758, VF0755, VF0298, VF0849, and VF0858) were present across groups but exhibited lower prevalence in the DO group.

Analysis of pathogen-host interactions using the PHI database (Figures 8C, D) revealed a higher relative abundance of factors associated with skin infections in the DO group, indicating a skewed microbial community potentially contributing to dandruff. At level 2, specific pathogen-host interactions were highlighted. The much higher levels of *Staphylococcus aureus* in the DO group indicate a connection to inflammation and infection, upset the balance of the skin microbiota. Conversely, the HO group exhibited increased levels of fungi (*Ustilago maydis* and *Candida albicans*), which may contribute to scalp health through competition with pathogenic organisms. The HN group exhibited a more balanced microbial profile, indicative of a healthier scalp environment.

### 3.8 Genomic plasticity and horizontal gene transfer

**Figure 9:**
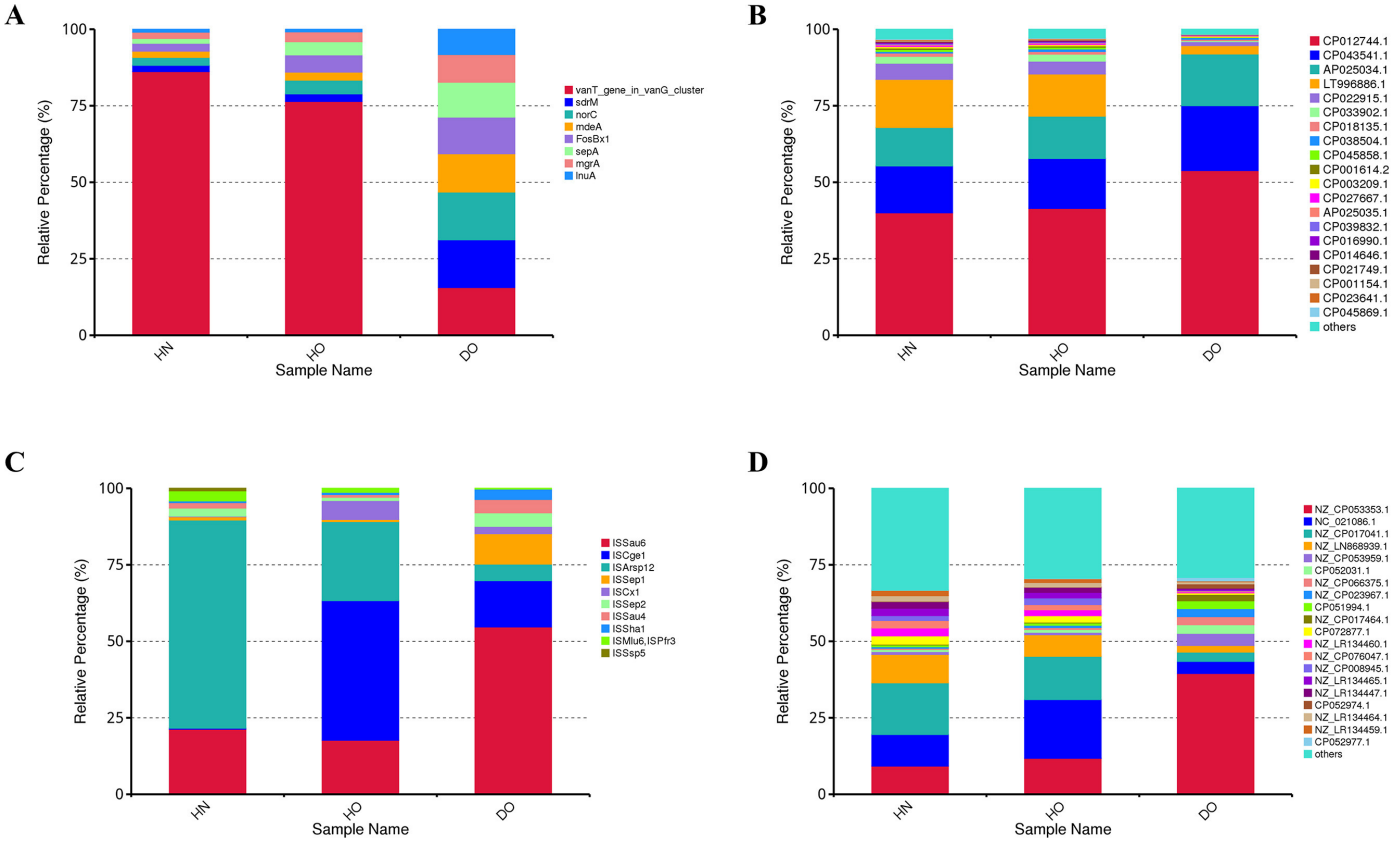
Resistance gene analysis in healthy and dandruff samples. Relative abundances of ARGs **(A)**, integrons **(B)**, insertion sequence elements **(C)**, and plasmids **(D)** shown by histograms.

Figures 9A–D show significant differences in the relative abundance of ARGs and mobile genetic elements (MGEs) among the three scalp samples groups. Figure 9A shows the relative abundance of key ARGs. The *vanT* gene (*vanG* cluster) consistently exhibits high relative abundance, particularly in healthy groups (76.41% in HO and 86.16% in HN of the total ARG pool), suggesting widespread potential for vancomycin resistance. Other ARGs (*sdrM, norC, mdeA, fosBx1, sepA, mgrA*, and *inuA*) account for the remaining 15–25% of the ARG pool. While these genes maintain similar proportions in the DO group (8.23–15.6%), with *vanT, sdrM*, and *norC* each representing ~15.6%, many are associated with *Staphylococcus aureus*, suggesting potential contributions to resistance mechanisms.

Figure 9B depicts the relative abundance of integrons. The integron CP012744.1 constitutes ~40–50% of the total integron pool, indicating its significant role in the microbiome's genetic landscape. This integron is significantly more abundant in the DO group (53.81%) compared to HN (40.01%) and HO (41.45%), suggesting an adaptive response to selective pressures associated with dandruff. In contrast, integron LT996886 is nearly five times more prevalent in the healthy groups, possibly reflecting a protective role in maintaining scalp health. The diverse integron composition further underscores the complexity of microbial interactions and resistance mechanisms.

Figure 9C shows the relative abundance of the top 10 insertion sequence (IS) elements. In HN samples, ISArsp12 (68.01%) and ISSau6 are most prevalent. ISSau6, linked to *Staphylococcus aureus*, is significantly more abundant in DO (54.71%) compared to HN (21.28%) and HO (17.67%), suggesting a microbial shift during dandruff development that may contribute to inflammation. ISCge1, primarily found in HO (45.65%), exhibits lower frequency in HN (0.36%) and DO (15.12%), suggesting a potential protective role. Other IS elements show subtle variations.

Figure 9D shows the relative abundance of plasmids. Plasmid NZ_CP053353.1, originating from *Staphylococcus aureus*, is significantly more abundant in DO (39.49%) compared to HN (9.24%) and HO (11.77%), suggesting a link to dandruff-associated microbial dynamics. Healthy groups show a relatively even distribution of plasmids NC_021086.1, NZ_CP017041.1, and NZ_LN868939.1 (36–40% of total plasmid pool), potentially indicating a more stable plasmid composition reflecting a balanced ecosystem. The remaining plasmids exhibit considerable diversity and variation across groups.

## 4 Discussion

Variations in sebum secretion can influence scalp conditions, leading to ecological imbalances that contribute to dandruff (Borda and Wikramanayake, 2015; Ro and Dawson, 2005; Yoon et al., 2021). To investigate the differential interaction mechanisms underlying different scalp conditions, we conducted an integrative omics study analyzing the composition and function of the scalp microbiome in individuals with and without dandruff, focusing on oily scalp conditions. Our research findings indicate a complex interplay of microbial communities, functional pathways, and genomic plasticity that contribute to the condition of dandruff pathogenesis.

Both amplicon and shotgun metagenomic sequencing demonstrated significant alterations in the scalp microbiome between dandruff-affected oily (DO) scalps and healthy oily (HO) and non-oily (HN) scalps. Amplicon sequencing indicated that the scalp microbiome is predominantly composed of three phyla, with substantial variations noted among the groups. It is important to note that the DO group displayed a heightened prevalence of *Firmicutes*, specifically of the genus *Staphylococcus*, which has been associated with inflammation linked to dandruff (Liu et al., 2018). In contrast, the genus *Cutibacterium* was found to be more prevalent in healthy groups, suggesting its potential protective role against scalp disorders (Rozas et al., 2021). The fungal community, primarily consisting of *Ascomycota* and *Basidiomycota*, exhibits no notable differences among the scalp samples. Nonetheless, it encompasses the genus *Malassezia*, recognized for its association with various skin disorders (Prohic et al., 2016). Our findings regarding the fungal community composition, specifically the relatively low abundance of *Malassezia* in DO group are not consistent with some previous studies (Grimshaw et al., 2019). This inconsistency may stem from methodological variations, differences in study populations (such as ethnicity and geographic location), or our limited sample size. The presence of *Malasseziales_gen_Incertae_sedis*, an unclassified genus within the *Malassezia* family in the DO group, can potentially lead to further research in determining *Malassezia* imbalance.

The results from our alpha and beta diversity analyses further emphasized that while the DO group shares a common microbial community with the healthy groups, distinct differences in abundance underline a complex community structure rather than the presence of a singular causative agent for dandruff. The constructed genus-level relationship network revealed unique interaction patterns among genera. For instance, the observed negative correlation between *Cutibacterium* and *Lawsonella* in the HN group indicates competitive dynamics influencing their ecological roles. In the HO group, *Pseudonocardia* emerged as a key component, suggesting its importance in shaping community dynamics, while the negative correlation between *Lawsonella* and *Pseudomonas* suggests competition that further distinguishes this group (Cosseau et al., 2016; Watanabe et al., 2021). Interestingly, the lack of interaction between *Staphylococcus* and both *Cutibacterium* and *Lawsonella* in the DO group implies niche differentiation, potentially enhancing community stability and resilience.

At the species level, shotgun metagenomic sequencing has offered supplementary insights. Species such as *Cutibacterium acnes, Malassezia restricta*, and *Staphylococcus aureus* align with prior research, indicating the presence of a shared microbial community among scalp samples (Soares et al., 2016; Grimshaw et al., 2019). This is particularly true for *Pseudomonas* sp., which appeared in greater numbers in dandruff samples, indicating its potential influence on scalp health homeostasis. One study also indicated that the relative abundance of *Pseudomonas* is associated with severe scalp psoriasis (Choi et al., 2022). Additionally, the increased abundance of *Escherichia coli* in the DO group is noteworthy. Although not a typical resident of the scalp microbiome, its elevated levels suggest potential translocation from hair or other sources, possibly contributing to inflammation or dysbiosis. One study has shown that *Escherichia coli* and *Pseudomonas aeruginosa* demonstrate distinct adherence patterns on hair surfaces, effectively colonizing without significantly affecting the hair shaft's surface morphology (Kerk et al., 2018).

These findings establish the groundwork for an in-depth examination of the functional profiling of these microbial communities, which may further clarify their roles in scalp health and disease. KEGG pathway analysis revealed a higher abundance of carbohydrate and amino acid metabolism, nucleotide and energy metabolism, and membrane transport in HN and HO samples, suggesting more active, adaptive microbiomes. In contrast, the dandruff-affected (DO) group showed reduced representation in these pathways, potentially indicating impaired nutrient uptake and metabolism linked to dysbiosis (Belizario et al., 2018).

Functional profiling via eggNOG highlighted increased DNA repair functions in the DO group, suggesting stress responses due to pathogen overgrowth, a characteristic of dysbiosis often seen in dandruff (Trueb et al., 2018; Polak-Witka et al., 2020). Conversely, HN samples displayed enhanced functions in transport and membrane biogenesis, reinforcing the idea that a balanced microbiome is crucial for maintaining scalp homeostasis and preventing inflammatory conditions, which include dandruff (Polak-Witka et al., 2020; Lousada et al., 2021).

Further insights from the CAZy database analysis indicated that the microbiota in HN samples demonstrates robust adaptability for utilizing a diverse range of carbohydrate substrates. This capability potentially supports skin health and enhances barrier function. Conversely, the DO group displayed a diminished capacity to degrade complex structural components of the stratum corneum, indicating possible deficiencies in nutrient acquisition essential for maintaining scalp integrity. The elevated abundance of AA and PL in healthy oily (HO) samples further suggests that a well-functioning scalp microbiome contributes positively to skin barrier integrity, aiding in the resolution of inflammation (Prescott et al., 2017). While Kruskal–Wallis on centroid distances provided a conservative test for beta diversity, future studies with larger cohorts should apply PERMANOVA to assess centroid shifts directly.

The analysis of virulence factors underscored their critical role in maintaining scalp health and their association with dandruff. Factors, including VF0317 and VF0302, which are associated with immune modulation and resistance to oxidative stress, were identified as being more abundant in individuals characterized as healthy. This suggested that higher levels of these factors contributed to a protective microbial environment against pathogens. Conversely, the reduced abundance of these factors in scalps affected by dandruff indicated a predominance of pathogenic bacteria, leading to increased inflammation and microbial dysbiosis. For instance, VF0842 appeared to exacerbate inflammation by promoting pathogenic colonization or triggering local immune responses. Additionally, other virulence factors like VF0738 and VF0758 were also observed to be less abundant in dandruff-affected scalps, further indicating a diminished capacity for protective functions.

Analysis from the PHI database corroborated these observations, revealing a higher prevalence of skin infection-associated factors in the DO group, particularly from *Staphylococcus aureus*. This association may contribute to inflammation and exacerbate dandruff symptoms. The healthy-oily scalp group (HO) showed a notable level of *Candida albicans*, potentially aiding the survival of *Staphylococcus aureus* by offering protection against antimicrobial agents via polysaccharide-mediated biofilm formation. This dynamic interplay suggests that while *Candida albicans* may provide defensive benefits against certain pathogens, it could also facilitate bacterial resilience within polymicrobial environments (Kong et al., 2016).

Additionally, our analysis of ARGs, integrons, Is elements, and plasmids identified significant differences between the sample groups, particularly highlighting increased genomic plasticity within the DO group. The presence of *Staphylococcus aureus*-associated ARGs—such as *sdrM*, and *norC*—in both healthy and dandruff-affected groups suggests that these microorganisms have evolved mechanisms to withstand environmental stresses, including antibiotic pressure (Anjum et al., 2024). Notably, the elevated abundance of ARGs and MGEs in the DO cohort indicates an increased potential for horizontal gene transfer, contributing to microbial instability and adaptability (Thomas and Nielsen, 2005). This rise in ARG prevalence, especially among *Staphylococcus* species, reflects selective pressures arising from antibiotic usage in the treatment of dandruff, which may inadvertently promote the emergence of antibiotic-resistant strains. Furthermore, the enhanced presence of plasmids, integrons, and diverse Is elements signifies the dynamic nature of the microbiome associated with dandruff, further amplifying its virulence and adaptability in response to therapeutic interventions (Bennett, 2008).

The gender-specific analysis, although not explicitly detailed in the results, is a critical strength of the study and needs further discussion in the context of observed differences. Hormonal variations between males and females could influence sebum production, pH, and immune responses, potentially impacting the structure and function of the scalp microbiome. An exploration of the gender-specific aspects of microbiome composition and function in dandruff will facilitate a more comprehensive understanding of the disease and pave the way for more customized therapeutic intervention approaches.

## 5 Limitations

While our research offers original insights, it is essential to recognize specific limitations. Although we applied FDR correction and LEfSe to address compositional bias, future studies should use modeling approaches (e.g., MaAsLin2) to integrate covariates. Furthermore, the study's design limits causal determination; therefore, longitudinal studies are needed to understand microbiome fluctuations and their relationship with dandruff progression. The restricted sample size (*n* = 7 per cohort for metagenomic examination) might impede the statistical strength and broader significance of the findings. Further research involving larger cohorts is essential to validate these results. Additionally, a comprehensive and integrative approach to determine microbial contributors with different gene functions is required to enhance understanding of how the microbiome can impact dandruff pathology. Finally, the functional predictions rely on bioinformatic analyses; experimental validation of predicted functions is necessary for complete confirmation.

## 6 Conclusion

This integrative omics study provides strong evidence that a dysbiotic scalp microbiome is central to the pathogenesis of dandruff, particularly in individuals with oily scalps. Our analysis reveals significant differences in microbial composition, virulence factors, and metabolic capacities between healthy and dandruff-affected scalps. The DO group showed signs of dysbiosis, including increased DNA repair functions and a higher prevalence of pathogenic bacteria, which may contribute to inflammation and exacerbate dandruff. In contrast, healthy scalps (HN and HO) displayed enhanced functions supporting skin health, such as carbohydrate metabolism and immune modulation. Additionally, the study highlights differences in genomic plasticity and antibiotic resistance genes in the dandruff-associated microbiome, suggesting its adaptability and potential for pathogenicity. This research underscores the importance of considering the entire scalp microbiome ecosystem, rather than focusing solely on species like *Malassezia restricta*. A balanced microbiome is crucial for preventing dandruff and other inflammatory conditions. Future studies should investigate the temporal dynamics of microbiome changes, validate functional predictions, and explore microbiome-targeted therapies to restore balance and alleviate dandruff symptoms. The integrated approach applied here offers a valuable framework for investigating other complex skin conditions and for developing more personalized and effective treatments.

## Data Availability

The original contributions presented in the study are publicly available. This data can be found here: https://www.ncbi.nlm.nih.gov, accession number PRJNA1199187.

## Supplementary material

Supplementary Figure S1 Taxonomic annotations by Krona for HN **(A)**, HO **(B)**, and DO **(C)** groups.

Supplementary Figure S2 Top 6 relative abundances of level 1 KEGG functional pathways among HN, HO, and DO groups.

Supplementary Table S1 Integrated summary of microbial taxonomic profiles, alpha diversity indices, genus-level differential abundance (*t*-test statistics for DO-vs-HN and HO-vs-DO comparisons), demographic metadata, and functional annotations across HN, HO, and DO groups, including: bacterial/fungal phylum (top 10) and genus (top 30) composition from 16S/ITS amplicon sequencing; metagenomic species-level genus rankings (top 10); DIAMOND functional alignment results; and alpha diversity metrics for all samples.
