# Supplementary figures and images for: Dysbiosis and genomic plasticity in the oily scalp microbiome: a multi-omics analysis of dandruff pathogenesis
Source: Front Microbiol. 2025 Jul 4;16:1595030. doi: 10.3389/fmicb.2025.1595030 (PMC12271121; doi:10.3389/fmicb.2025.1595030)

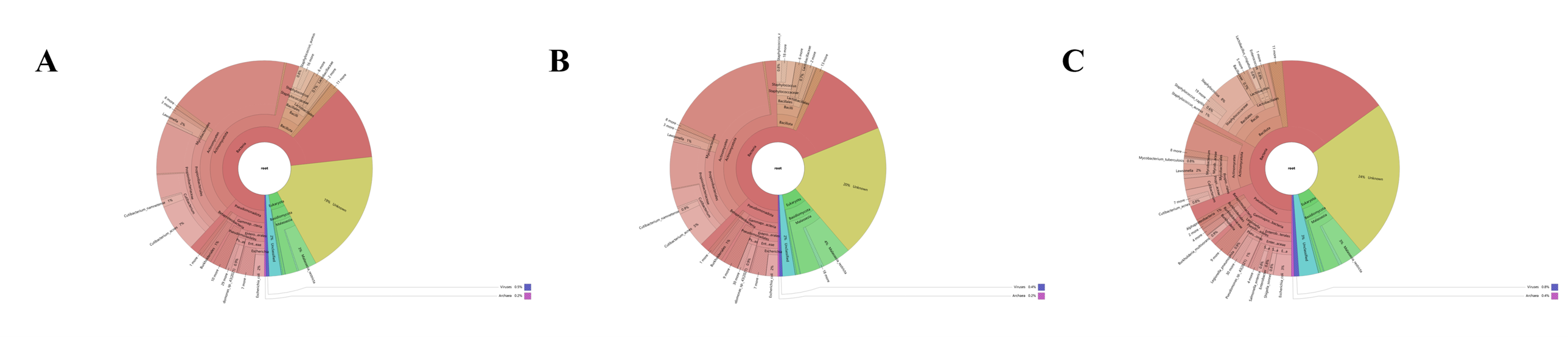

Supplement: Supplementary Figure S1 — Taxonomic annotations by Krona for HN (A), HO (B), and DO (C) groups. [file Image_1.tif]

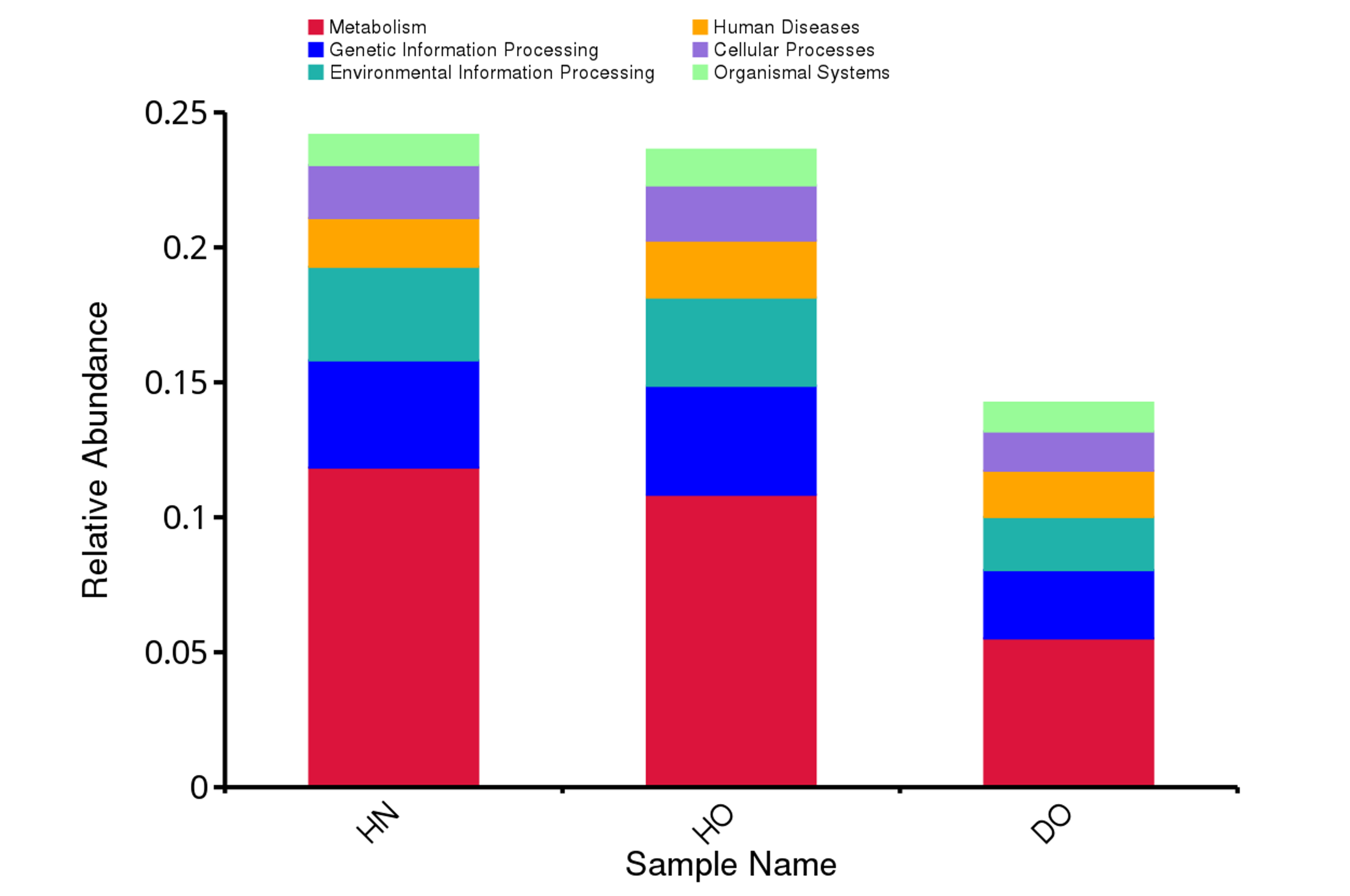

Supplement: Supplementary Figure S2 — Top 6 relative abundances of level 1 KEGG functional pathways among HN, HO, and DO groups. [file Image_2.tif]
